# Interspecific Interactions between *Phragmites australis* and *Spartina alterniflora* along a Tidal Gradient in the Dongtan Wetland, Eastern China

**DOI:** 10.1371/journal.pone.0053843

**Published:** 2013-01-16

**Authors:** Yue Yuan, Kaiyun Wang, Dezhi Li, Yu Pan, Yuanyuan Lv, Meixia Zhao, JinJin Gao

**Affiliations:** 1 Key Lab of Urbanization and Ecological Restoration, East China Normal University, Shanghai, China; 2 National Field Observation and Research Station in the Tiantong Forest Ecosystem, East China Normal University, Ningbo, Zhejiang, China; 3 Department of Environmental Science, East China Normal University, Shanghai, China; The Ohio State University, United States of America

## Abstract

The invasive species *Spartina alterniora* Loisel was introduced to the eastern coast of China in the 1970s and 1980s for the purposes of land reclamation and the prevention of soil erosion. The resulting interspecific competition had an important influence on the distribution of native vegetation, which makes studying the patterns and mechanisms of the interactions between *Spartina alterniora* Loisel and the native species *Phragmites australis* (Cav.) Trin ex Steud in this region very important. There have been some researches on the interspecific interactions between *P. australis* and *S. alterniora* in the Dongtan wetland of Chongming, east China, most of which has focused on the comparison of their physiological characteristics. In this paper, we conducted a neighbor removal experiment along a tidal gradient to evaluate the relative competitive abilities of the two species by calculating their relative neighbor effect (RNE) index. We also looked at the influence of environmental stress and disturbance on the competitive abilities of the two species by comparing interaction strength (I) among different tidal zones both for *P. australis* and *S. alterniora*. Finally, we measured physiological characteristics of the two species to assess the physiological mechanisms behind their different competitive abilities. Both negative and positive interactions were found between *P. australis* and *S. alterniora* along the environmental gradient. When the direction of the competitive intensity index for *P. australis* and *S. alterniora* was consistent, the competitive or facilitative effect of *S. alterniora* on *P. australis* was stronger than that of *P. australis* on *S. alterniora*. The interspecific interactions of *P. australis* and *S. alterniora* varied with environmental conditions, as well as with the method used, to measure interspecific interactions.

## Introduction

One of the major goals of ecology is to understand the forces that generate patterns in natural communities [1]. Ecologists have focused on competition as a crucial process for community organization [2], but facilitation may also be critical in some plant assemblages [3]. The intensity and direction of interspecific interactions may be affected by environmental conditions as well as the species being studied [4]–[6]. A number of experiments have studied interspecific interactions along natural gradients, including competition along a productivity gradient [2], [7]–[9] and along a stress and disturbance gradient [5].

Invasive plants are one of the most serious threats to native species assemblages and have been responsible for the degradation of natural habitats worldwide [10]. Wetlands appear to be especially vulnerable to invasions. Many wetland invaders form monotypes, which alter habitat structure, lower biodiversity (both the number and “quality” of species), change nutrient cycling and productivity (often increasing both), and modify food webs [11]. Thus, it is important to understand the interactions between invasive species and native species under different stress and disturbance conditions, as such understanding might be helpful for the effective conservation and management of wetland ecosystems.

Salt marshes are ideal for examining plant interspecific interactions along gradients of stress and disturbance [1]. Tidal flooding establishes a strong non-resource-based stress and disturbance gradient across a marsh landscape. The stress gradient is produced by anoxic, waterlogged soil that decreases from the seaward edge to the terrestrial border of the marsh, and the disturbance gradient is produced by the direct effects of wave action, which removes biomass more rapidly from exposed shores than from sheltered shores [3]. Additionally, the height of the shore above sea level is often used as a qualitative bulk parameter in salt marshes [12].

*Phragmites australis* (Cav.) Trin. ex Steud. (common reed) and *Spartina alterniflora* Loisel (smooth cordgrass) are two well-known invasive salt marsh species [13], [14] in different regions. *Phragmites australis*, a salt marsh species native to the east coast of China, is aggressively invading salt marshes along the Atlantic coast of North America [15], [16]. *Spartina alterniflora*, a grass native to the tidal salt marshes of the southeastern USA, has invaded extensive areas along the Chinese and European coasts and has increased dramatically in distribution and abundance [17], [18]. In both cases, the non-native grass is thought to degrade the habitat value of the marsh for wildlife [19], exerting a significant influence on wetland community structure via mechanisms such as decreasing plant diversity [15] and reducing the extent of habitat function for trophic support across a broad range of consumer species [20], [21].

Our study site is a typical tidal marsh in the Chongming Dongtan wetland in the Yangtze River estuary in east China. The marsh plant community at the site is presently dominated by two clonal perennial species: an indigenous species, *Phragmites australis*, and an alien species, *Spartina alterniflora*. *Spartina alterniflora* was introduced to the eastern coast of China for the purposes of land reclamation and the prevention of soil erosion in the 1970s and 1980s [22]. Since that time, the species has spread rapidly and replaced *Scirpus mariqueter,* a native species that previously occupied the low tidal zone [23]. In the same period, the abundance of *P. australis* within the study area has declined annually. The reduction of *P. australis* can be attributed to multiple causes including land reclamation and the introduction of alien species. Our aim in studying the interactions between *P. australis* and *S. alterniflora* and the implications of these interactions for community structure is to shed light on the extent to which *S. alterniflora* is responsible for the decline of *P. australis*.

There are dozens of indices with which to measure competition intensity [24]–[26]. The relative competitive index (RCI), which compares the performance of a target plant grown mixed with neighbors and grown in isolation, is one of the most widely used indices [26]. The relative neighbor effect (RNE) is an improvement on the RCI. This index is symmetric around zero and constrained by +1 (competition) and –1 (facilitation), so it can be used to estimate facilitative interactions that RCI cannot [27]. In field experiments, neighbors’ biomass varies as a function of the capacity of each habitat to support growth. The RNE does not consider differences in capacity among habitats, although increased crowding can also change the competitive influence of neighbors as a group without altering the competitive abilities of individual plants. Therefore, simply comparing RNEs (which cannot distinguish between the per-unit effect and the effect of crowding on neighbor biomass) is not adequate for understanding the competitive abilities of different species under various conditions. In 2007, Wilson proposed two competitive indices to address this problem: the effect of relative crowding (Dr) and interaction strength (I). He defined the effect of relative crowding (Dr) as the ratio of the abundance of neighbors to the potential size of the target plant and interaction strength (I) as the ratio of the change in the performance of a target plant grown mixed with neighbors vs. grown in isolation to the abundance of neighbors [26]. Using index I, the competitive abilities of individual plants under varied conditions can be compared.

**Figure 1:**
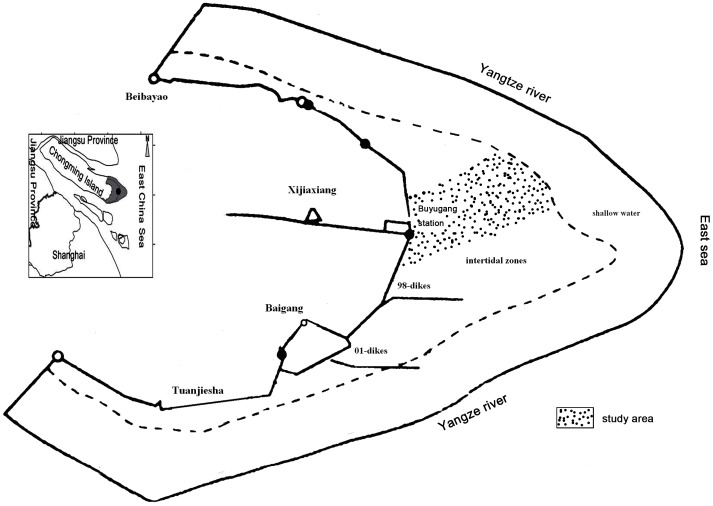
The location of our study area in the Chongming Dongtan Nature Reserve, Shanghai.

At the Buyugang protection station in the Dongtan wetland within the Yangtze River estuary in Chongming, Shanghai, eastern China, there is an environmental gradient from the seaward edge of the wetland to dike number 98 (Fig. 1). Soil salinity and inundation were the primary physical factors influencing the growth of the dominant plant species *P. australis* and *S. alterniflora* in different zones. However, we found that the distributions of the two species formed a mosaic pattern across almost the entire intertidal zone. This pattern clearly suggests that eco-physiological tolerances alone might be insufficient to explain the pronounced zonation of the two species across the tidal gradient, and the interactions between the two species might be different in different intertidal habitats. Findings from the natural soil salinity gradient suggest that as salt stress increases, plant distributions in coastal marshes will be less influenced by competition and increasingly influenced by facilitation [4], [28], [29]. Patterns in marsh plant communities clearly represent a delicate balance between competitive and facilitative interactions. To assess the existing and future ecological relationships between the two species within different intertidal habitats, we examined and compared the interspecific interactions between the species along the tidal gradient. Our intention was to use controlled species removals in natural sympatric stands to test the hypothesis that the intensity and direction of interspecific interactions between the invasive species *S. alterniflora* and native species *P. australis* will change with the environmental gradient and species identity [3], [9], [29], [30]. We aimed to address the following questions: What is the interspecific interaction (competition or facilitation) between *P. australis* and *S. alterniflora* in different intertidal habitats? In other words, how does the interspecific relationship vary along the tidal gradient? Which physiological characteristics may contribute to the competitive abilities of the two species?

## Materials and Methods

### Ethics Statement

The field investigation conducted in this study was approved by the Chongming Dongtan Wetland Nature Reserve. Migratory birds are protected in the study area, and we did our best to avoid the bird migration season in the process of the experiment. No protected species were sampled or disturbed.

### Study Species

*Spartina alterniflora* is a perennial rhizomatous C_4_ grass [31]. Its shoots can grow up to 1–3 m in height with hard leaves 30–90 cm long. *Spartina alterniflora* spreads through both clonal propagation by rhizome and sexual reproduction by seed [32]. Its ramets are active from spring to autumn. Most of the old ramets die during the winter, whereas young ramets that appear in autumn stop growing and survive the winter months. *Spartina alterniflora* also has the ability to reproduce sexually and can produce as many as 600 seeds per inflorescence [22].

*Phragmites australis*, which is native to the Dongtan wetland, is a perennial rhizomatous C_3_ grass [31]. The shoots can reach approximately 4 m in height. Although *P. australis* is able to reproduce sexually, it relies primarily on vegetative growth for recruitment. The rhizome systems of *P. australis* are perennial, tough, rich in fiber, and can spread extensively [33], [34].

### Study Site

**Figure 2:**
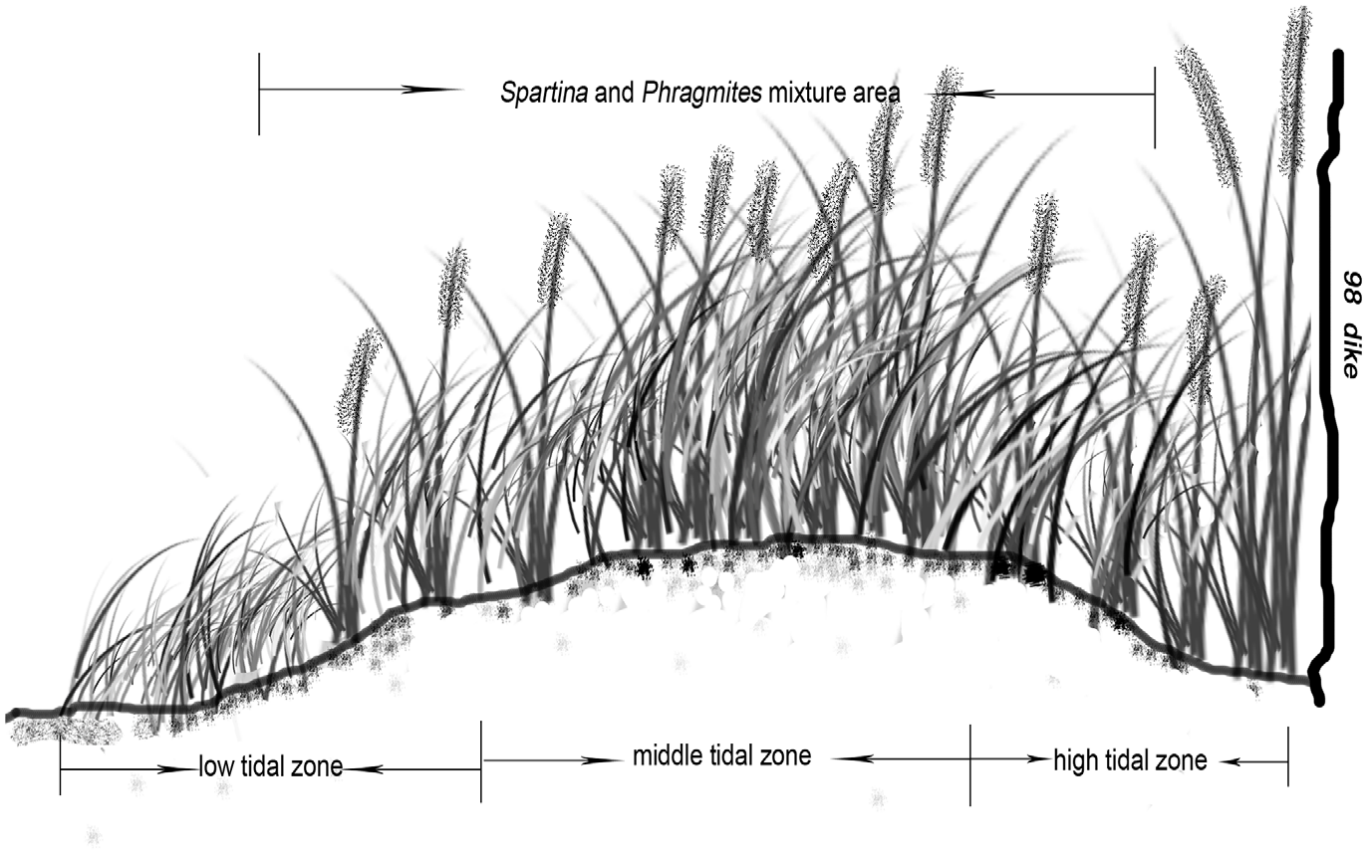
The distribution of *P. australis* and *S. alterniflora* in the research area along the environmental gradient.

Field studies were conducted at the Buyugang protection station of the Dongtan wetland (31°25′–31°38′N, 121°50′–122°05′E), which is located at the east end of Chongming Island in the Yangtze River estuary. The Yangtze River is ranked third, fourth, and fifth among the world’s rivers with regard to its length, annual sediment flux, and water discharge to the sea, respectively. Chongming Island is the world’s largest alluvial island, covering 1200 km^2^. It increases in size by approximately 500 ha annually through the deposition of sand, silt, and mud by the Yangtze River. The Dongtan wetland is now a natural reserve in China. Tides in this area are semi-diurnal. As a tidal marsh, the Dongtan wetland is very productive and affected by the periodic tides. Due to the repeated flooding, the Dongtan wetland has developed distinct intertidal zones, including a coastal shallow-water zone below the mean low-water line [35]. The wetland is 8 km wide at its maximum width in the intertidal zone, with the uppermost 2.5 km covered by marsh vegetation (Fig. 1). Within the intertidal zone, the water and salt contents in the soil vary as a function of elevation. In the high, middle, and low tidal zones, the water content in the soil is approximately 34%–35%, 27%–32%, and 33%–39%, respectively, and the content of NaCl is approximately 14–25 ppt, 25–34 ppt, and 11–21 ppt, respectively [36]. The salt marsh in the study area exhibits obvious vegetation zonation and displays a successional sequence in the following order: uncovered mudflats, *Spartina*-dominated community, *Spartina* and *Phragmites* mixture, *Phragmites*-dominated community. In the Buyugang area, located in the northeast of the Dongtan wetland, *Phragmites australis* and *Spartina alterniflora* typically co-occur as dominant species. The *Phragmites australis* and *Spartina alterniflora* mixture covers approximately three-fourths of the total area distributed across all three tidal zones (Fig. 2).

### Environmental Gradient and Vegetation

On April 10^th^, 2010, we first measured the elevation of each tidal zone relative to the Wusong Tidal Height datum using an optical level gauge. Next, thirty 10 cm×10 cm×10 cm soil quadrats were placed 100 m apart along line transects within each tidal zone, and soil samples were collected by shovel from each quadrat. On August 10^th^, 2010, fifty 1×1 m^2^ plots were established randomly within the study area. The abundance of ramets for *P. australis* and *S. alterniflora* was recorded, as well as the height of each ramet in each plot. We considered each ramet as an “individual” of the species in our measurements. In clonal plants, a ramet can be treated as a relatively and potentially independent “individual” [37]. The direct competition among relatively independent ramets of different species in a community should be considered a primary constraint on the growth of different species, although resource integration among ramets within the clone of a species might also exist to some extent. If the genet of a clonal plant was considered an “individual” in studying interspecific interactions in a natural situation, in most cases, each plot would contain only one or two “individuals” belonging to one or two species. Thus, the measurement of interspecific interaction would become confused. For this reason, we considered a ramet an individual for the purposes of measuring the abundance of the two species and studying their interspecific interactions. The importance values of the two species were then calculated. The importance value [38] was expressed as (C_r_+H_r_)/2, where C_r_ is the relative coverage of the species and H_r_ is the relative height of the species. These characteristics of *P. australis* and *S. alterniflora* were compared using an analysis of variance.

Samples from the leaves, stems, and roots of *P. australis* and *S. alterniflora* were also collected. We established three 1×1 m^2^ *S. alterniflora* plots and three 1×1 m^2^ *P. australis* plots within each tidal zone. Then, the aboveground biomass of *P. australis* and *S. alterniflora* was removed using scissors and separated into leaves and stems. Additionally, 100 cm×100 cm×30 cm soil samples were carefully removed with a shovel, and the roots of the two species were separated from the soil using a flushing method. All samples were taken to the laboratory as soon as possible and stored under refrigeration.

In the laboratory of the East China Normal University, soil salinity (DDS-11A conductivity meter) and soil total nitrogen (N) and total phosphorus (P) (Skalar Santt flow injection analyzer) were measured, as were the non-structural carbohydrates (NSC) (Anthrone colorimetry), N and P contents, and the N:P ratio (Skalar Santt flow injection analyzer) of different parts of *P. australis* and *S. alterniflora* (including the leaves, stems, and roots). Significant differences in soil salinity, total N, and total P among the different tidal zones were tested via an analysis of variance. NSC, the N and P contents, and the N:P ratio of all three organs of *P. australis* and *S. alterniflora* were compared among different tidal zones. Significant differences were tested using an analysis of variance.

### Neighbor Removal Experiment

From April to June, the growth of *P. australis* and *S. alterniflora* is slow, and their population densities and culm heights are low. From July to October, the growth of *P. australis* and *S. alterniflora* becomes rapid, and their competitive intensity usually reaches its peak at this time. After October, the growth of *P. australis* and *S. alterniflora* slows once again and the culms wither gradually. On July 10^th^, 2011, neighbor removal experiments were conducted in mixed *Spartina-Phragmites* plots within every tidal zone. The physical conditions of each plot in the same tidal zone were nearly identical. Three treatments were conducted: a control treatment, a *Spartina* removal treatment in which all of the aboveground parts of *S. alterniflora* were cut, and a *Phragmites* removal treatment in which all of the aboveground parts of *P. australis* were cut. Every month, we used scissors to remove the aboveground biomass of *P. australis* or *S. alterniflora*. The belowground parts of the two species intertwined and were difficult to separate relatively intact, so only the aboveground biomass of the neighbors was removed. To avoid the influence of intraspecific competition on interspecific competition as much as possible, 20 1×1 m^2^ plots in which *S. alterniflora* was dominant and 20 1×1 m^2^ plots in which *P. australi*s was dominant were subjectively chosen at each site. In *S. alterniflora*-dominated plots, 10 plots were chosen randomly as controls, and the remaining 10 plots underwent the *S. alterniflora* removal treatment. The same approach was used for 20 *P. australis*-dominated plots. In October, the center of each quadrat (10×10 cm^2^) was harvested; tillers were sorted to the species level, counted, and measured (height). The aboveground biomass of each species was oven-dried and weighed.

### Competition Intensity

First, we used the relative neighbor effect index (RNE) [23] to measure the interspecific interactions of *P. australis* and *S. alterniflora*. The RNE was calculated as follows:
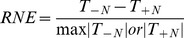
(1)where T_-N_ is the performance of the target species in the absence of neighbors and T_+N_ is the performance of the target species in the presence of neighbors [15].

In our experiment, the performance of the target species was defined as the relative growth rate per day (RGR) and the number of newly produced tillers (TNT). The RGR [9] was calculated as follows:
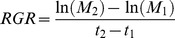
(2)where M_2_ is the shoot mass at the end of the experiment, M_1_ is the shoot mass at the beginning of the experiment, and t_2_–t_1_ is the number of days of the experiment.

Similarly, we defined TNT as follows:
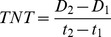
(3)where D_2_ is tiller density at the end of the experiment, D_1_ is the tiller density at the beginning of the experiment, and t_2_–t_1_ is the number of days of the experiment. The RNE was calculated for RGR:
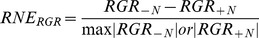
(4)and for TNT:



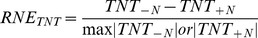
(5)In addition, we further calculated the interaction strength (I) of the two species in different tidal zones.
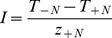
(6)where z_+N_ is the abundance of neighbors surrounding the target plant, T_-N_ is the performance of a target plant grown without neighbors and T_+N_ is the performance of a target plant grown with neighbors [26]. Similarly, we also defined the performance of target plants from two perspectives: RGR and TNT. The interaction strength (I) was calculated for RGR as follows:



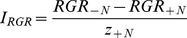
(7)Similarly, the interaction strength (I) was calculated for TNT as follows:
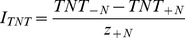
(8)


To calculate the mean RGR of the target plants, we needed to determine their biomass before the treatments. To estimate this, we established an additional 40 plots that were similar to the other experimental plots. The center of each quadrat (10×10 cm^2^) was harvested at the start of the experiment. Tillers were sorted to the species level, counted, and measured (height). The aboveground biomass of each species was oven-dried and weighed.

We first compared the RNE index values of *P.australis* and *S. alterniflora* in the different tidal zones using an analysis of variance. Next, the change in the intensity of the interspecific interactions along the tidal gradient was analyzed by comparing I among the different tidal zones separately for *P. australis* and *S. alterniflora*. Significant differences in I among tidal zones were tested using an analysis of variance. Finally, correlations between I and environmental factors were calculated for both *P. australis* and *S. alterniflora*.

## Results

### Population Characteristics in the Study Area and Environmental Gradient

In the Dongtan salt marsh, both *P. australis* and *S. alterniflora* are dominant species; few other species exist. The mean importance value per plot of the two species was not significantly different within the study area (P>0.05) (table 1). The mean height of *P. australis* was significantly higher than that of *S. alterniflora* (*P*<0.01), and the mean density and biomass per plot of *S. alterniflora* were significantly higher than those of *P. australis* (*P*<0.01) (table 1).

**Table 1:** Population characteristics in the study area (n = 50 plots) (mean±SE).

Species	Density (No.m^−2^)	Height (cm)	Biomass (g/m^2^)	Important value
*P. australis*	36.65±3.41	140±7.5	565.95±35.12	0.46±0.00
*S. alterniflora*	72.21±4.3	100±7.5	1628.98±240.55	0.54±0.01

A notable environmental gradient existed in the study site. The relative elevation of the middle tidal zone was higher than both the high and low tidal zones (p<0.01). Soil salinity increased and the N content decreased along the tidal gradient from the high tidal zone to the low tidal zone (p<0.01), but the P content did not change notably between the three intertidal zones (p>0.05) (table 2).

**Table 2:** Physical characteristics in the different tidal zones (n = 30 plots) (mean±SE).

Location	Relative elevation (m)	Soil salinity (ppt)	N % (mg/g)	P % (mg/g)
High tidal zone	2.61±0.06	18.54±3.05	1.6±0.174	0.40±0.08
Middle tidal zone	19.22±2.45	22.14±3.97	1.26±0.10	0.47±0.06
Low tidal zone	1.55±0.03	32.59±6.75	0.95±0.06	0.38±0.06

### Neighbor Removal Experiment

#### The responses of *P. australis* and *S. alterniflora* to neighbor removal

In all three tidal zones, the mean RGR and mean TNT of *S. alterniflora* were positive in both the control and neighbor removal treatments. The mean RGR of *S. alterniflora* was significantly (P<0.05) greater in plots with neighbors removed than in plots with neighbors left intact in all three tidal zones. The mean TNT of *S. alterniflora* was only significantly (P<0.05) greater in plots with neighbors removed than in plots with neighbors left intact in the low tidal zone.

The mean RGR and mean TNT of *P. australis* were negative or positive with different treatments. The mean RGR was significantly (P<0.05) higher in plots with neighbors removed than in plots with neighbors left intact in the low tidal zone and significantly (P<0.05) lower in plots with neighbors removed than in plots with neighbors left intact in the high tidal zone. The mean TNT of *P. australis* was significantly (P<0.05) lower in plots with neighbors removed than in plots with neighbors left intact in all three tidal zones (table 3).

**Table 3:** The relative growth rate per day (RGR) and the number of newly produced tillers per day (TNT) responses of *S. alterniflora* and *P. australis* to neighbor removal in different tidal zones (mean±SE).

Species		High tidal zone	Middle tidal zone	Low tidal zone
*P. australis*	RGR_N+_(g.g^−1^.d^−1^)	0.1±0.02	−0.05±0.01	0.03±0.00
	RGR_N-_ (g.g^−1^.d^−1^)	−0.02±0.02	−0.06±0.05	0.13±0.04
	TNT_N+_ (no.d^−1^)	0.2±0.05	0.3±0.07	0.05±0.03
	TNT_N-_ (no.d^−1^)	−0.13±0.03	0.18±0.06	−0.42±0.06
*S. alterniflora*	RGR_N+_(g.g^−1^.d^−1^)	0.31±0.03	0.25±0.13	0.23±0.01
	RGR_N-_ (g.g^−1^.d^−1^)	0.39±0.03	0.27±0.02	0.24±0.15
	TNT_N+_ (no.d^−1^)	0.06±0.0	0.04±0.01	0.07±0.04
	TNT_N-_ (no.d^−1^)	0.05±0.01	0.02±0.03	0.11±0.03

RGR_N+_ represents the relative growth rate per day (RGR) when neighbors present. RGR_N-_ represents the relative growth rate per day (RGR) when neighbors absent. TNT_N+_ represents the number of the newly produced tillers per day (TNT) when neighbors are present. TNT_N-_ represents the number of the newly produced tillers per day (TNT) when neighbors are absent.

#### Interspecific interactions between *P. australis* and *S. alterniflora*

**Figure 3:**
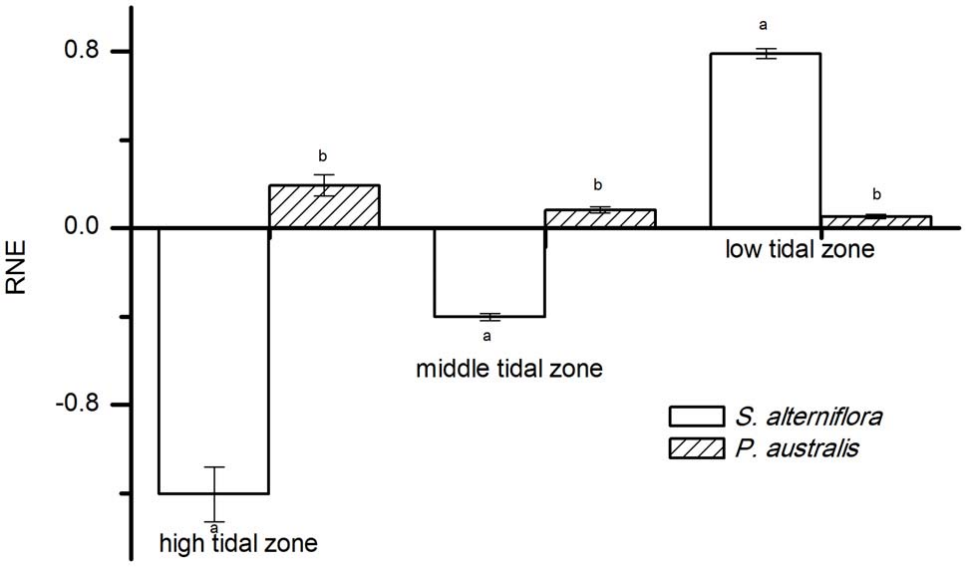
The relative neighbor effect (RNE) of *P. australis* and *S. alterniflora* in different tidal zones. The performance of target plants was measured by the relative growth rate per day (RGR). Different letters indicate significant differences.

**Figure 4:**
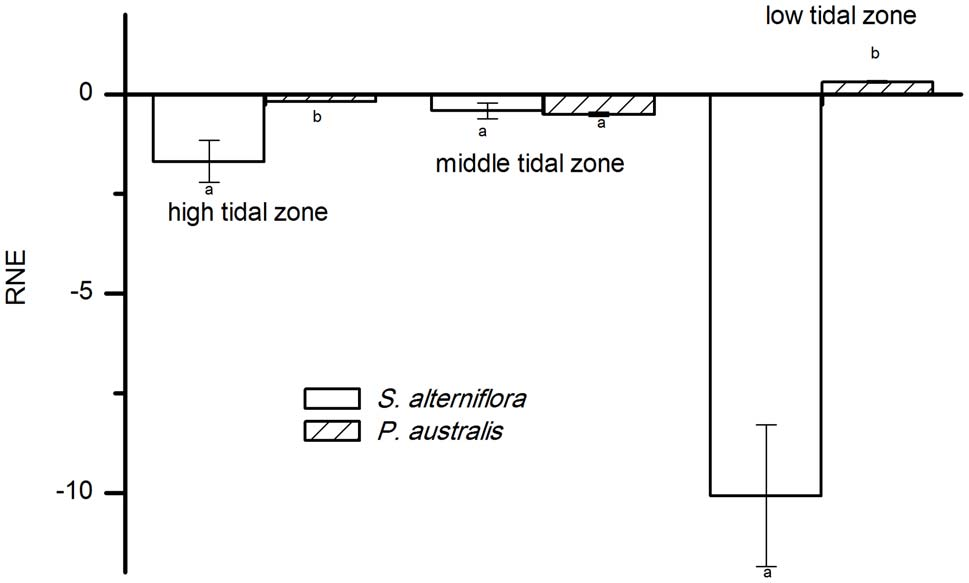
The relative neighbor effect (RNE) of *P. australis* and *S. alterniflora* in different tidal zones. The performance of target plants was measured by the number of newly produced tillers per day (TNT). Different letters indicate significant differences.

We estimated the interspecific interactions of the two species by calculating their RNE values (See Fig. 3 and Fig. 4). The RNE_RGR_ represented the effect of the interactions on the growth of the target ramet and the RNE_TNT_ represented the effect of the interactions on the survival and spread of the target ramet.

In the high and middle tidal zones, the RNE_RGR_ of *P. australis* was positive and the RNE_RGR_ of. *S. alterniflora* was negative. In the low tidal zones, the RNE_RGR_ was positive for both *P. australis* and *S. alterniflora*, and the effect of *S. alterniflora* was stronger than that of *P. australis* (p<0.01) (Fig. 3).

The RNE_TNT_ was negative for both *P. australis* and *S. alterniflora* in the high and middle tidal zones. The effect of *S. alterniflora* was stronger than that of *P. australis* (p<0.01) in the high tidal zone, but the competitive effects of the two species were not significantly different in the middle tidal zone (p>0.01). In the low tidal zone, the RNE_TNT_ of *P. australis* was positive and the RNE_TNT_ of *S. alterniflora* was negative (Fig. 4).

#### Interspecific interactions related to the tidal gradient

In this study, changes in the competitive ability of neighbors, which can be described by interaction strength (I), were compared along the tidal gradient for both *P. australis* and *S. alterniflora.* Similarly, I_RGR_ represents the effect of the interactions on the growth of the target ramet and I_TNT_ represents the effect of the interactions on the survival and spread of the target ramet.

**Figure 5:**
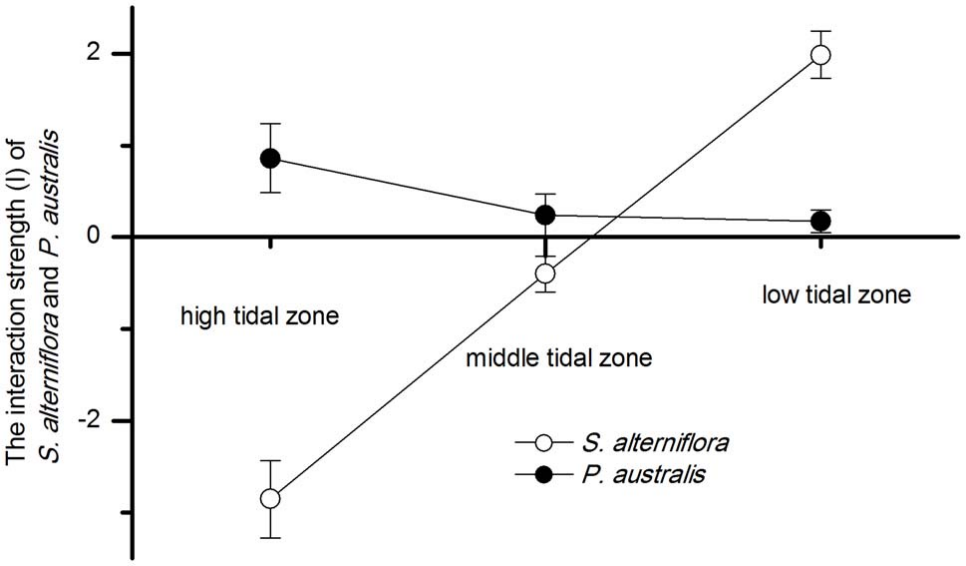
The interaction strength (I) of *P. australis* and *S. alterniflora* along the tidal gradient. The performance of targets was measured by the relative growth rate per day (RGR).

The I_RGR_ of *P. australis* was positive and that of *S. alterniflora* was negative in both the high and middle tidal zones. The I_RGR_ was positive for both *P. australis* and *S. alterniflora* in the low tidal zone, and the I_RGR_ of *S. alterniflora* on the target plants was stronger than that of *P. australis*. The I_RGR_ of *P. australis* on *S. alterniflora* decreased over the tidal gradient from the high tidal zone to the low tidal zone. The I_RGR_ of *S. alterniflora* on *P. australis* increased over the same tidal gradient (Fig. 5). A significant positive correlation was found between the I_RGR_ of *S. alterniflora* and the soil salinity (r = 0.94, p<0.001), and a significant negative correlation was found between the I_RGR_ of *S. alterniflora* and the N content (r = −0.98, p<0.001). In contrast, a significant negative correlation was found between the I_RGR_ of *P. australis* and the soil salinity (r = −0.75, p<0.05), and a significant positive correlation was found between the I_RGR_ of *P. australis* and the N content (r = 0.92, p<0.05) (table 4).

**Table 4:** Correlation of soil characteristics and the interaction strength (I)of *S. alterniflora* and *P. australis*.

	Relative elevation	Soil salinity	N %	P %
I_RGR_ of *S. alterniflora*	−0.363	−0.756*	0.926**	−0.218
I_RGR_ of *P. australis*	−0.072	0.949**	−0.989**	−0.156
I_TNT_ of *S. alterniflora*	−0.772*	0.701	−0.731	−0.527
I_TNT_ of *P. australis*	0.991**	−0.365	0.046	0.749

I_RGR_ represents the interaction strength (I) that was calculated for the relative growth rate per day. I_TNT_ represents interaction strength (I) that was calculated for the number of the newly produced tillers per day.

* *P*<0.05;

** *P*<0.01.

**Figure 6:**
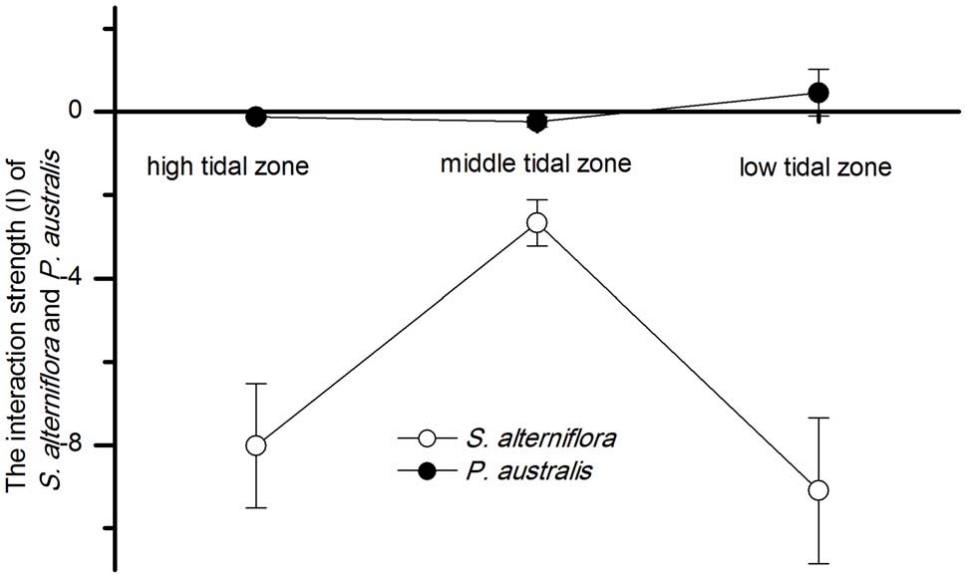
The interaction strength (I) of *P. australis* and *S. alterniflora* along the tidal gradient. The performance of targets was measured by the number of newly produced tillers per day (TNT).

The I_TNT_ of *P. australis* was positive and that of *S. alterniflora* was negative in the low tidal zone. The I_TNT_ of both *P. australis* and *S. alterniflora* was negative in the high and middle tidal zones, and the I_TNT_ of *S. alterniflora* on the target plants was stronger than that of *P. australis*. The I_TNT_ of *P. australis* on *S. alterniflora* was close to zero in all three tidal zones, and the competitive effect of *S. alterniflora* on *P. australis* was strongest in the low tidal zone and weakest in the middle tidal zone (Fig. 6). A significant correlation was observed between I_TNT_ and the relative elevation for both *P. australis* (r = −0.67, p<0.05) and *S. alterniflora* (r = 0.99, p<0.001) (table 4).

### Some Related Physiological Characteristics

**Figure 7:**
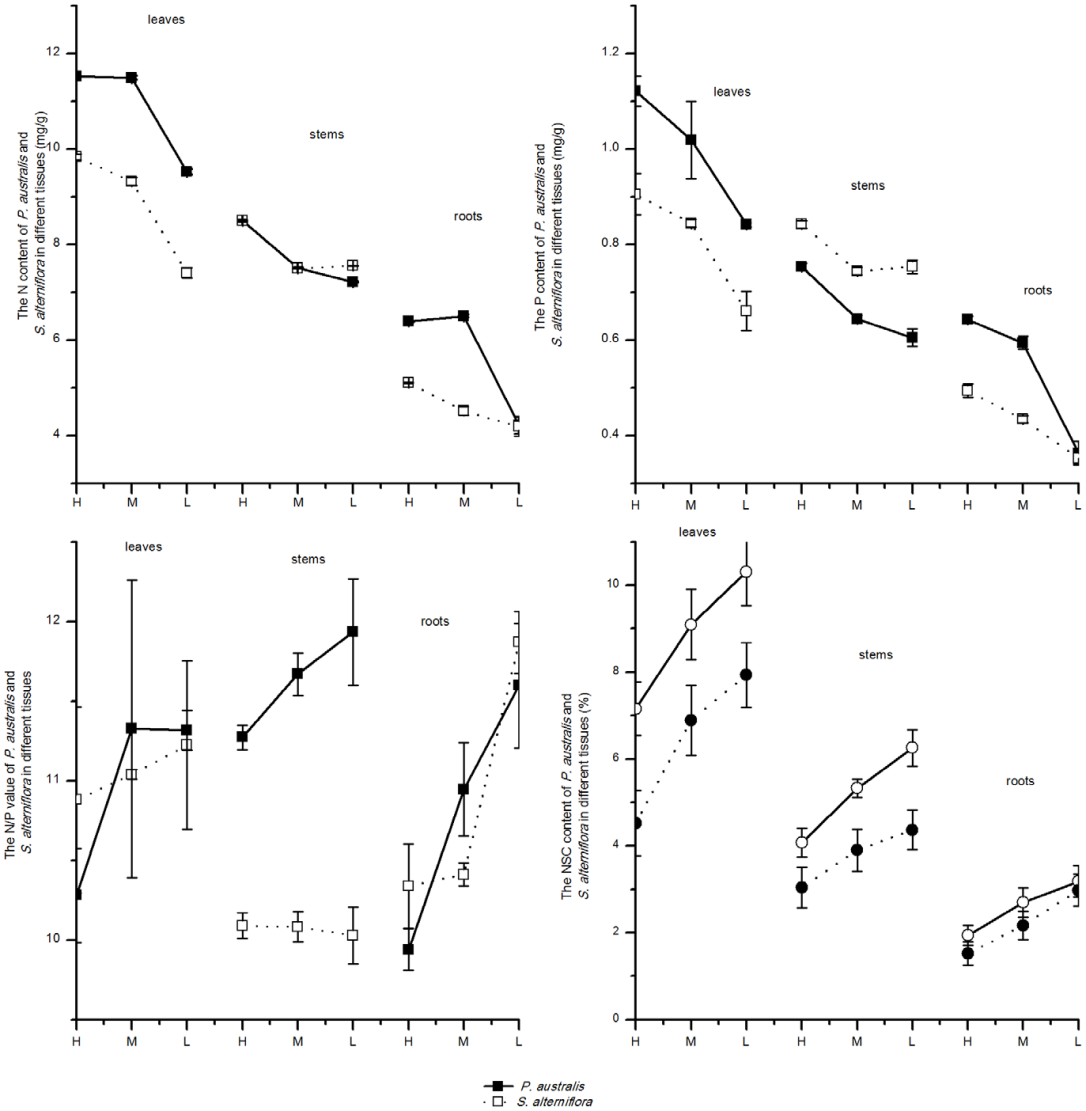
The physiological characteristics of *P. australis* and *S. alterniflora* along different tidal gradient. Physiological characteristics measured included non-structural carbohydrates (NSC), nitrogen (N) and phosphorus (P) contents, and N:P in different organs (leaves, stem, and roots) of the two species.

The NSC content in all organs (leaves, stems, and roots) of *S. alterniflora* was significantly higher than that in *P. australis* (p<0.05) in all three tidal zones. The N and P contents in the leaves and roots of *P. australis* were significantly higher than those of *S. alterniflora* (p<0.05), and the P content in the stems of *P. australis* was significantly lower than that of *S. alterniflora* in all three tidal zones (p<0.05). The N:P ratios in the leaves and roots of *P. australis* and *S. alterniflora* differed among tidal zones, and the N:P ratio in the stems of *P. australis* was significantly higher than that of *S. alterniflora* stems in all three tidal zones. Along the tidal gradient (from high to low), both the NSC content in different organs (leaves, stems, and roots) and the N:P ratio of *P. australis* and *S. alterniflora* increased, and the N and P contents in the different organs of the two species decreased. The NSC content and N:P ratio increased more quickly in *S. alterniflora* than in *P. australis*, and the N:P ratios of the two species were less than 15, which indicates that N was the limiting element for both species [6] (Fig. 7).

## Discussion

*Phragmites australis* is spreading into North American coastal marshes and has become a dominant species in marsh tidal wetlands of North America [16], [39], whereas in the Yangtze River estuary of China and in northern European brackish marshes, *Spartina alterniflora* is spreading quickly and appears to have a competitive advantage compared to native species in these areas [19], [40]. The two situations are in sharp contrast, and it is difficult to explain why each species can successfully invade the other’s native habitat [41]. However, when we consider the ecophysiological characteristics of the two species, particularly with regard to their adaptations to soil salinity and elevation, their performance in non-native habitats is understandable. Ecophysiological differences can shift the competitive advantage from one species to another in different environmental conditions [18], [19], [42].

In the previous studies [36], [41], [43]–[48] conducted in our study area, some reports indicated that the relative competitive ability of *S. alterniflora* was significantly greater than that of *P. australis* [45], [46], [48], which might explain the rapid spread of *S. alterniflora* over *P. australis* in some habitats [36]. Other reports indicated that the relative competitive dominance of *S. alterniflora* and *P. australis* was a function of different conditions [44]. However, some researchers argued that the invasion of *S. alterniflora* facilitated the spread of *P. australis* [43]. Our results suggest that interactions between *P. australis* and *S. alterniflora* in the saltmarsh can vary from competitive to facilitative along the tidal gradient. The competitive abilities of *P. australis* and *S. alterniflora* changed between tidal zones. A variety of interspecific interactions between *P. australis* and *S. alterniflora* in different stress and disturbance conditions can support these conclusions.

Although some studies have concluded that facilitative interspecific interactions increase with increasing stress and disturbance along an environmental gradient [28], [29], [49], other studies have shown that interspecific competition is greatest in the purportedly most stressful and disturbed zone [30]. Our results showed that the changes in interspecific interactions along the environmental gradient were influenced by species identity. The competitive effect of *P. australis* on *S. alterniflora* decreased along the gradient from the high tidal zone to the low tidal zone, whereas the effect of *S. alterniflora* on *P. australis* shifted from facilitative to competitive along the same tidal gradient. Moreover, one of the findings of this study is that most interactions between the two species were facilitative for asexual production (tiller production) but competitive or neutral for biomass. This may occur, e.g., if one species can protect the other from wave action to facilitate ramet production but the two species compete for resources (light, soil, etc.) for biomass accumulation. This result was similar to earlier research by Franks, who found that the interactions between *Uniola paniculata* and *Iva imbricata* in dunes were facilitative for survival but competitive or neutral for biomass [30]. Additionally, Levine reported that in a riparian community in California, *Carex nudata* competed with associated species by reducing their biomass but facilitated neighbors by protecting them from mortality during winter disturbances [50]. In contrast, our study described the survival of the target species in terms of clonal production in different treatments rather than by survival in transplant experiments. That is, we studied ramet survival. Goldberg and Novoplansky and Schupp have performed relevant theoretical work on survival facilitation and biomass competition [51], [52].

The greater salt tolerance of *S. alterniflora* compared with *P. australis* might be due to the ability of the former species to use Na^+^ and NSC for osmotic adjustment in shoots [53]. Our results also indicated that the NSC of *S. alterniflora* was greater than that of *P. australis* in all three tidal zones and increased more quickly than that of *P. australis* along the tidal gradient from a high tidal zone to a low tidal zone. Because *P. australis* has a competitive ability to use dissolved organic nitrogen (DON), the increased soil N content enhanced the overall competitive ability of *P. australis* [54]. Additionally, in the habitats with lower salinities, *P. australis* produced more shoots per gram of rhizome tissue than *S. alterniflora* did [19]. Koerselman and Meuleman found that the N:P ratio of vegetation directly indicates the nature of nutrient limitation at the community level [55]. They also put forward some critical N:P ratio values, according to which the limitation of plant growth by either N or P or both can be judged [55]–[57]. Based on these results, we can theoretically analyze the relationships of the growth of *S. alterniflora* and *P. australis* with soil N and P along the tidal gradient. In our study, the N:P ratio of *S. alterniflora* increased more quickly than that of *P. australis* along the tidal gradient from the high tidal zone to the low tidal zone. N limitation for *S. alterniflora* was weaker than that for *P. australis* in the low tidal zone. This reduced N limitation serves as an additional competitive advantage for *S. alterniflora* in this zone.

The problem of invasive species and their control is one of the most pressing applied issues in ecology today [58]. The control and eradication of *S. alterniflora* and *P. australis* have been studied widely in their respective invasive areas [59]–[62]. In general, control of *P. australis* by increasing flooding depth, salinity and/or sulfide concentrations has been considered [59]. Clipping vegetation at the early florescence stage and the integrated technique of cutting plus waterlogging are more efficient for controlling the invasive plant *S. alterniflora* [60], [61]. Our results may provide some guidance for managers using biological methods to control invasive plants. Different control measures should be implemented based on the competitive abilities of the two species in different tidal zones.

In the high tidal zone, the competitive ability of *P. australis* is high, and it has a competitive dominance over *S. alterniflora* because grazing disturbance has increased the soil N content in this zone, which is advantageous to the growth and spread of *P. australis* [19], [63], [64]. These results are similar to studies of *P. australis* in North America showing that shoreline development reduces soil salinities and increases nitrogen availability, both of which promote the invasion of *P. australis* [15], [21]. In addition, *S. alterniflora* replaced *P. australis* in the relatively low-lying and higher salinity plots in high tidal zones and constructed creekbank levees that may be colonized by *P. australis* [65]. In this way *S. alterniflora* facilitates the invasion of *P. australis* into the central marsh. This indicates that *S. alterniflora* does not have a competitive advantage as an invasive species and does not require control in the high tidal zone. In the middle tidal zone, the competition between *P. australis* and *S. alterniflora* was especially intense, and they formed a mosaic of patches. The competitive abilities of *P. australis* and *S. alterniflora* were similar in this zone, and dominance depended on the development of the salt marsh. *P. australis* might have a genetic competitive advantage over *S. alterniflora* because of its strong I. Therefore, over the long term, *P. australis* could be more successful if there were no other disturbances. To promote the spread of *P. australis* in the mixed community and to control the invader *S. alterniflora*, some artificial measures should be taken to accelerate the natural process. For example, *S. alterniflora* can be manually removed, and favorable conditions for the growth of *P. australis* can be created. In the low tidal zone, flood stress and disturbance is generally severe and soil salinity is relatively high, so the competitive ability of *S. alterniflora* was higher and it dominated in this tidal zone [3], [47], [66], whereas the competitive effect of *P. australis* on *S. alterniflora* reached its lowest point. Thus, it is difficult to replace *S. alterniflora* with *P. australis* in this zone, and managers should focus their attention on the middle tidal zone to control the further spread of *S. alterniflora*. Presently, *S. alterniflora* is nearly the only species that can occupy the otherwise bare shoreline habitats of the Dongtan wetland and contributes to siltation and the protection of shoreline areas. In other words, *S. alterniflora* plays unique and positive roles in these special areas. If *S. alterniflora* can be kept in these places sustainably and its invasion into middle and high tidal zones can be prevented, we believe that *S. alterniflora* need not to be thoroughly eradicated from the Dongtan wetland.

In conclusion, *Phragmites australis* is spreading into North American coastal marshes that are experiencing reduced salinities at the same time that *Spartina alterniflora* is spreading into northern European brackish marshes that are experiencing increased salinities as land use patterns change on the two continents [19]. In China, situations are more complicated. On the one hand, grazing disturbance has caused the soil N content to increase, which is advantageous to the growth and spread of *P. australis* [19], [63], [64]. On the other hand, reclamation has greatly reduced the population size of *P. australis* in natural conditions. Thus, the invasion of *S. alterniflora* has been indirectly influenced by human activity [67], [68]. Where reclamation efforts have largely reduced the area of *P. australis*, *S. alterniflora* can become rampant. However, according to our results, *P. australis* has greater competitive ability (higher I value) and may invade the *S. alterniflora* zone under natural conditions. Moreover, the Dongtan wetland of Chongming is rapidly growing through the deposition of sand, silt and mud carried by river runoff. With the continuous sedimentation and the increase in elevation [69], the relationship between *P. australis* and *S. alterniflora* will change, especially with the rising elevation of the present low and middle tidal zones. The habitat conditions of the present middle tidal zone will become more similar to the present high tidal zone, which would be advantageous to the spread of *P. australis*. *S. alterniflora* would gradually retreat from the presently occupied zones under such a scenario due to the rising elevation of these zones but would still remain a dominant species in the habitats near the shoreline. If *S. alterniflora* can be sustainably maintained in these originally bare shoreline areas where it can play a protective role, it need not be completely removed from this area. However, its invasion into the middle and high tidal zones needs to be prevented. Established populations there should be removed.
